# Left atrial enlargement and high uric acid level are risk factors for left atrial thrombus or dense spontaneous echo contrast in atrial fibrillation patients with low to moderate embolic risk assessed by CHA_2_DS_2_-VAS_C_ score

**DOI:** 10.3389/fcvm.2023.937770

**Published:** 2023-07-03

**Authors:** Chao-Di Tan, Juan-Zhang Liu, Yu-Ping Zheng, Zong-jian Li, Shu-Xian Zhou

**Affiliations:** Department of Cardiology, Guangzhou Key Laboratory of Molecular Mechanism and Translation in Major Cardiovascular Disease, Sun Yat-sen Memorial Hospital, Sun Yat-sen University, Guangzhou, China

**Keywords:** atrial fibrillation, uric acid, left atrium, thrombus, dense spontaneous echo contrast, CHA_2_DS_2_-VASc score

## Abstract

**Aims:**

To investigate the correlation and predictive value of left atrial diameter and blood uric acid levels with the occurrence of left atrial thrombus or dense spontaneous echo contrast in atrial fibrillation patients with low to moderate CHA_2_DS_2_-VASc scores.

**Methods and results:**

A total of 849 inpatients diagnosed with atrial fibrillation who had low to moderate CHA_2_DS_2_-VASc scores and complete transesophageal echocardiography were included in this study. Among them, 66 patients had left atrial thrombus or dense spontaneous echo contrast. When different models were used to correct other known risk factors, acid levels and abnormal left atrial diameter were identified as additional risk factors for left atrial thrombus or dense spontaneous echo contrast. The incidence of left atrial thrombus or dense spontaneous echo contrast was higher in patients with abnormal serum uric acid levels than in the control group (12.4% vs. 5.6%, *p* < 0.05), and this difference persisted after correcting the baseline data with propensity score matching (10.6% vs. 4.1%, *p* < 0.05). Abnormal left atrial diameter was another risk factor suggested by regression analysis, with an increased incidence of left atrial thrombus or dense spontaneous echo contrast in the abnormal left atrial diameter group compared to the control group, both before (18.0% vs. 3.5%, *p* < 0.05) and after (15.5% vs. 5.2%, *p* < 0.05) propensity score matching. The best predictive value was obtained by adding both abnormal serum uric acid levels and abnormal left atrial diameter.

**Conclusion:**

Left atrial enlargement and high uric acid levels increase the risk of left atrial thrombus or dense spontaneous echo contrast in atrial fibrillation patients with low to moderate CHA_2_DS_2_-VASc scores.

## Introduction

Atrial fibrillation (AF) is a common type of rapid supraventricular arrhythmia characterized by uncoordinated atrial excitation and consequently ineffective atrial contraction. Ischemic stroke and embolism of circulatory arteries are major complications in patients with AF, and the risk of ischemic stroke is 5 times higher among these patients. If cardioembolic stroke occurs, it may be followed by high mortality rates (up to 20%) and disability rates (60%) (1–4).

A left atrial thrombus (LAT) may occur if the duration of AF exceeds 48 h. The presence of spontaneous echo contrast (SEC) is an important marker of thrombus formation. Previous studies have shown both dense SEC (Fatkin class 3+ and 4+) and LAT to be prerequisites for thromboembolism in patients with AF, as both have been independently associated with thromboembolic events in these patients (5–9).

Previous studies have shown that hypertension, diabetes, female sex, congestive heart failure, advanced age, left atrial enlargement, renal insufficiency, N-terminal brain natriuretic peptide precursor, left atrial appendage morphology, and blood emptying velocity are independent risk factors for stroke in patients with AF (10–12). Serum uric acid (SUA) level has also been reported to be associated with LAT (13). In addition, endothelial dysfunction (decreased endothelial nitric oxide synthase activity and expression) and changes in blood components (platelet activation, inflammatory response) have been associated with thrombosis in patients with AF (14, 15).

The CHA_2_DS_2_-VASc score is currently guideline-recommended for stratifying the risk of thromboembolism in patients with AF. Men with a CHA_2_DS_2_-VASc scor ≥2 and women with a CHA_2_DS_2_-VASc score ≥3 are considered high-risk patients for whom oral anticoagulant therapy is recommended, and patients with a CHA_2_DS_2_-VASc score of 0 or 1 (including women with a score of 2) are considered to be at low to moderate risk of stroke. However, previous studies have shown that a certain percentage of patients with AF who were deemed to be at low to moderate risk still developed thromboembolic stroke, suggesting that unknown factors were contributing to the development of LAT and SEC besides the known risk factors included within the CHA_2_DS_2_-VASc score. There are few studies about the risk factors of stroke in patients with AF who are otherwise classified as low risk. In one such study, Yan et al. proposed that a high Lp(a) plasma level and left atrial dilatation might be independent risk factors of thrombotic events for AF patients with a low CHA_2_DS_2_-VASc score (16). In a different study, Yao et al. found that an elevated plasma homocysteine level increases the risk of LA/LAA thrombus in AF patients with a low CHA_2_DS_2_-VASc score (17). In addition, LAA anatomy could be a risk factor (18). Recently, an increasing amount of research has focused on the relationship between SUA and thromboembolism risk. However, it remains unclear whether SUA can offer sufficient predictive value for LAT/dense SEC in AF patients with a low to moderate CHA_2_DS_2_-VASc score.

Identifying patients who are at high risk of LAT or dense SEC from among those who are typically defined as low to moderate risk according to their CHA_2_DS_2_-VASc score has important clinical implications. To that end, we retrospectively analyzed the clinical data of 849 AF patients with low to moderate risk CHA_2_DS_2_-VASc scores to discern risk factors not included in the CHA_2_DS_2_-VASc score that may point to LAT or dense SEC in an effort to help further identify patients at high risk of thromboembolism.

## Methods

### Study population

Inpatients diagnosed with AF who also had complete transesophageal echocardiography (TEE) performed at Sun Yat-sen Memorial Hospital of Sun Yat-sen University from January 2007 to July 2019 were included in this study. Exclusion criteria were as follows: (1) rheumatic mitral stenosis, post mitral valve repair, or post-mechanical or biological valve replacement; (2) congenital heart disease; (3) severe infectious or autoimmune disease; (4) severe hepatic or renal insufficiency; (5) hyperthyroidism; (6) malignancy; (7) coagulation disorders; (8) those with psychiatric abnormalities; and (9) high CHA_2_DS_2_-VASc scores. Clinical data on gender, age, body mass index (BMI), anteroposterior LA diameter (LAD), left ventricular ejection fraction (LVEF), serum uric acid (SUA), hypertension, diabetes mellitus, stroke, peripheral vascular disease, heart failure, and history of gout were collected. Anteroposterior LAD ≥4 cm was defined as LA enlargement (abLAD). Serum uric acid >420 μmol/L was defined as hyperuricemia (abSUA). Patients were divided into a LAT/dense SEC group and a control non-LAT/dense SEC group according to the presence of both LAT and dense SEC.

### Assessment of CHA_2_DS_2_-VASc score

The CHA_2_DS_2_-VASc score was assessed for congestive heart failure (CHF), hypertension, diabetes mellitus, vascular disease (including peripheral vascular disease and myocardial infarction), age (1 point for ages 65–74 years and 2 points for ages ≥75 years), gender (1 point for women), and history of previous stroke/TIA. A score equal to 0 for men or 1 for women was considered low risk, 1 for men or 2 for women was considered moderate risk, and ≥2 for men or ≥3 for women was considered high risk. A total of 849 patients with low- to moderate-risk CHA_2_DS_2_-VASc scores were included in this study.

### Transesophageal echocardiography (TEE)

All patients with AF routinely undergo TEE 48h before ablation or cardioversion. LAT was defined as an echogenic image in the left atrium that is well-defined, homogeneous in density, and different from the density of adjacent myocardial tissue. SEC was defined as a TEE finding that shows cloudy, swirling, or pre-thrombotic echogenicity in the left atrium. Dense SEC was defined by a Fatkin classification of 3+ and 4+ according to the Fatkin classification of 5 levels of severity (5).

### Statistical analysis

Continuous variables were expressed as mean ± standard deviation, and independent sample *t*-tests were used for comparison between groups. Categorical variables were expressed as frequencies and percentages. Chi-square tests or Fisher's exact method tests were used for comparison between groups. Binary logistic regression analysis was used to screen for risk factors. All statistical tests were performed using SPSS 25.0.

Imbalanced baseline information data were adjusted by using a 1:1 propensity score match (PSM) without replacement. Propensity score matching was used to achieve balance in the baseline exposure group. Propensity scores were obtained using a logistic regression model. The variables used for matching were specifically reported in corresponding tables. The width of the caliper value used was equal to the log standard deviation of a propensity score of 0.02. The degree of balance between the baseline variables was assessed by standardized differences, with a standard deviation of ≤0.1indicating a high level of balance. PSM was performed using R3.6.3.

Receiver operating characteristic (ROC) analysis was used to evaluate the predictive effect of each variable on LAT/dense SEC. ROC analysis was performed using SPSS 25.0. A *p-*value <0.05 was considered statistically significant.

### Baseline information

A total of 849 patients were included in this study, including 31 with LAT and 35 with dense SEC. The LAT/dense SEC group had a lower LVEF; higher creatinine, SUA, LAD, and LVDD values; and a higher proportion of abSUA, abLAD, CHF, and alcohol consumption. The difference in CHA_2_DS_2_-VASc scores between the two groups was not statistically significant. The clinical and demographic characteristics of the patients are presented in Table 1.

**Table 1:** Comparison of baseline information between the LAT/dense SEC and non-LAT/dense SEC groups.

Characteristics	LAT/dense SEC group (*n* = 66)	Non-LAT/dense SEC group (Control) (*n* = 783)	*p*-value
Age (years)	58.05 ± 8.45	55.57 ± 10.03	0.052
Female	21 (31.8%)	281 (35.9%)	0.507
BMI (kg/m^2^)	25.33 ± 3.57	24.60 ± 3.30	0.085
Current smoking	22 (33.3%)	188 (24.0%)	0.092
Current alcohol drinking	10 (15.2%)	55 (7.0%)	0.017
Hypertension	23 (34.8%)	222 (28.4%)	0.263
Diabetes	3 (4.50%)	30 (3.80%)	0.737
CHF	5 (7.60%)	11 (1.40%)	0.005
Coronary artery disease	6 (9.10%)	83 (10.6%)	0.701
Vascular disease	0	5 (0.60%)	1.000
Creatinine (μmol/L)	89.45 ± 19.00	83.66 ± 19.07	0.018
eGFR (ml/min/1.73 m^2^)	76.72 ± 22.86	81.96 ± 24.27	0.091
Urine protein	0	12 (2.6%)	1.000
D-Dimer (mg/L FEU)	0.40 ± 0.56	0.42 ± 2.28	0.958
SUA (μmol/L)	434.80 ± 108.70	377.93 ± 93.74	<0.001
abSUA	34 (51.5%)	232 (29.6%)	<0.001
LVEF (%)	61.80 ± 9.25	66.95 ± 7.60	<0.001
LAD (mm)	42.97 ± 7.33	36.43 ± 5.33	<0.001
abLAD	43 (65.2%)	211 (26.9%)	<0.001
LVDD (mm)	50.77 ± 6.10	48.19 ± 4.54	0.001
CHA_2_DS_2_-VASc score	1.02 ± 0.67	0.85 ± 0.72	0.066
Antiplatelet	16 (24.2%)	130 (16.6%)	0.114
Anticoagulant	11 (16.7%)	96 (12.3%)	0.300
β-blocker	20 (30.3%)	165 (21.1%)	0.081
ACEI/ARB	7 (10.6%)	72 (9.2%)	0.705
Amiodarone	2 (3.0%)	68 (8.7%)	0.109

Data are presented as *n* (%) or mean ± SD. BMI, body mass index; CHF, chronic heart failure; SUA, Serum uric acid; LVEF, left ventricular ejection fraction; LAD, left atrial diameter; LVDD, left ventricular end diastolic diameter; abLAD, Anteroposterior LAD ≥4 cm; abSUA, Serum uric acid >420 μmol/L; LAT, left atrial thrombus; SEC, spontaneous echo contrast; ACEI, Angiotensin Converting Enzyme Inhibitor; ARB, Angiotensin Receptor Blocker.

### Binary logistic regression analysis of risk factors

Binary logistic regression analysis was used to screen for risk factors other than those included within the CHA_2_DS_2_-VASc score (Table 2). Of the included variables, abSUA, abLAD, current alcohol consumption, and LVDD were not included in the final score. Different models were used to correct other known risk factors. In model 1, after correction for age, sex, hypertension, diabetes, and CHF, we found that abSUA and abLAD were risk factors for LAT/dense SEC. In model 2, abSUA and abLAD were identified as risk factors for LAT/dense SEC after correction for age, sex, hypertension, diabetes, CHF, current alcohol consumption, creatinine, LVEF, LVDD, and CHA_2_DS_2_-VASc. In model 3, abSUA and abLAD were found to be risk markers for LAT/dense SEC after adjusting for current alcohol consumption, creatinine, LVEF, LVDD, and CHA_2_DS_2_-VASc. Overall, abSUA and abLAD were both found to be risk factors for LAT/dense SEC, so further analysis was done to corroborate these findings.

**Table 2:** Association between risk factors and the presence of LAT/dense SEC.

	Model 1		Model 2		Model 3	
	*p*-value	OR (95% CI)	*p*-value	OR (95% CI)	*p*-value	OR (95% CI)
abSUA	0.018	1.886 (1.113–3.195)	0.033	1.792 (1.048–3.066)	0.045	1.725 (1.012–2.942)
abLAD	<0.001	5.251 (3.025–9.114)	<0.001	4.503 (2.564–7.910)	<0.001	4.663 (2.655–8.190)
LVEF	–	–	0.001	0.952 (0.925–0.979)	0.002	0.958 (0.932–0.985)
CHF	0.037	3.357 (1.074–10.493)	–	–	–	–
Age	–	–	0.020	1.037 (1.006–1.070)	–	–

OR, odds ratio; CI, Confidence Interval; abSUA, Serum uric acid >420 μmol/L; abLAD, Anteroposterior LAD ≥4 cm; LVEF, left ventricular ejection fraction; LVDD, left ventricular end diastolic diameter; CHF, chronic heart failure.

Model 1: age, sex, hypertension, diabetes and CHF were adjusted.

Model 2: age, sex, hypertension, diabetes, CHF, current alcohol drinking, creatinine, LVEF, LVDD and CHA_2_DS_2_-VASc were adjusted.

Model 3: current alcohol drinking, creatinine, LVEF, LVDD and CHA_2_DS_2_-VASc were adjusted.

### abSUA is a risk factor for LAT/dense SEC

Because regression analysis suggested abSUA as a risk factor for LAT/dense SEC, further analysis was performed. The incidence of LAT/dense SEC was found to be higher in patients with abSUA than in the control group (12.4% vs. 5.6%, *p* < 0.05), and this difference persisted after correcting the baseline data with PSM (10.6% vs. 4.1%, *p* < 0.05) (Table 3).

**Table 3:** Comparison between abSUA and normal patients (PSM).

	Before matching			After matching		
	abSUA *N* = 274	Control *N* = 575	*p*-value	abSUA *N* = 224	Control *N* = 224	*p*-value
Age (years)	55.01 ± 10.30	56.12 ± 9.75	0.128	55.30 ± 10.47	55.54 ± 9.79	0.805
Female	54 (19.7%)	248 (43.1%)	<0.001	52 (23.2%)	46 (20.5%)	0.493
BMI (kg/m^2^)	25.65 ± 3.46	24.18 ± 3.15	<0.001	25.13 ± 3.30	25.04 ± 3.04	0.773
Current smoking	94 (34.3%)	116 (20.2%)	<0.001	66 (29.5%)	68 (30.4%)	0.836
Current alcohol drinking	30 (10.9%)	35 (6.1%)	0.013	22 (9.8%)	21 (9.4%)	0.873
Hypertension	93 (33.9%)	152 (26.4%)	0.024	70 (31.3%)	71 (31.7%)	0.919
Diabetes	16 (5.8%)	17 (3.0%)	0.042	13 (5.8%)	8 (3.6%)	0.264
CHF	8 (2.9%)	8 (1.4%)	0.126	4 (1.8%)	3 (1.3%)	0.703
CAD	28 (10.2%)	61 (10.6%)	0.862	21 (9.4%)	23 (10.3%)	0.751
Vascular disease	2 (0.7%)	3 (0.5%)	0.660	1 (0.4%)	2 (0.9%)	1.000
Creatinine (μmol/L)	90.49 ± 18.41	81.07 ± 18.71	<0.001	88.62 ± 18.46	88.13 ± 17.06	0.770
eGFR (ml/min/1.73 m^2^)	83.87 ± 25.17	80.44 ± 23.65	0.053	83.40 ± 26.16	81.42 ± 24.76	0.413
LVEF (%)	64.82 ± 8.56	67.41 ± 7.23	<0.001	65.88 ± 7.47	66.01 ± 7.63	0.865
LAD (mm)	38.47 ± 5.92	36.22 ± 5.53	<0.001	37.59 ± 5.28	36.96 ± 5.37	0.212
abLAD	109 (39.8%)	141 (24.5%)	<0.001	76 (33.9%)	65 (29.0%)	0.263
LVDD (mm)	49.29 ± 5.05	47.96 ± 4.51	<0.001	48.66 ± 4.58	48.53 ± 4.50	0.759
CHA_2_DS_2_-VASc score	0.77 ± 0.68	0.91 ± 0.73	0.007	0.77 ± 0.71	0.72 ± 0.66	0.410
LAT/dense SEC	34 (12.4%)	32 (5.6%)	<0.001	27 (12.1%)	14 (6.3%)	0.033

abSUA, abnormal serum uric acid; BMI, body mass index; CHF, congestive heart failure; CAD, coronary artery disease; eGFR, estimated glomerularfiltrationrate; LVEF, left ventricular ejection fraction; LAD, left atrial anteroposterior diameter; LVDD, left ventricular end diastolic diameter; LAT, left atrial thrombosis; SEC, spontaneous echo contrast; PSM, propensity score matching. Age, gender, BMI, current smoking, current alcohol drinking, hypertension, diabetes, CHF, CAD, vascular disease, LVEF, LAD, LVDD and eGFR were used to 1:1 PSM.

### abLAD is a risk factor for LAT/dense SEC

abLAD was another risk factor suggested by the regression analysis, with an increased incidence of LAT/dense SEC in the abLAD group compared to the control group, both before (18.0% vs. 3.5%, *p* < 0.05) and after (15.5% vs. 5.2%, *p* < 0.05) PSM was performed (Table 4). In conclusion, abSUA and abLAD were both found to be associated risk factors for LAT/dense SEC.

**Table 4:** Comparison between abLAD and normal patients (PSM).

	Before matching			After matching		
	abLAD *N* = 250	Control *N* = 599	*p*-value	abLAD *N* = 174	Control *N* = 174	*p*-value
Age (years)	56.84 ± 8.55	55.31 ± 10.43	0.028	56.87 ± 8.67	56.40 ± 9.44	0.632
Female	71 (28.4%)	231 (38.6%)	0.005	55 (31.6%)	52 (29.9%)	0.727
BMI (kg/m^2^)	25.95 ± 3.51	24.11 ± 3.09	<0.001	25.22 ± 3.47	25.37 ± 3.13	0.661
Current smoking	78 (31.2%)	132 (22.0%)	0.005	44 (25.3%)	51 (29.3%)	0.400
Current alcohol drinking	26 (10.4%)	39 (6.5%)	0.052	14 (8.0%)	19 (10.9%)	0.360
Hypertension	102 (40.8%)	143 (23.9%)	<0.001	61 (35.1%)	62 (35.6%)	0.911
Diabetes	10 (4.0%)	23 (3.8%)	0.912	6 (3.4%)	10 (5.7%)	0.306
CHF	11 (4.4%)	5 (0.8%)	0.001	3 (1.7%)	1 (0.6%)	0.623
CAD	21 (8.4%)	68 (11.4%)	0.201	14 (8.0%)	12 (6.9%)	0.683
Vascular disease	1 (0.4%)	4 (0.7%)	1.000	0	1 (0.6%)	1.000
Creatinine (μmol/L)	86.18 ± 18.24	83.25 ± 19.42	0.041	84.94 ± 17.98	86.82 ± 18.37	0.337
eGFR (ml/min/1.73 m^2^)	84.39 ± 22.88	80.37 ± 24.64	0.027	82.35 ± 23.12	82.91 ± 27.09	0.836
LVEF (%)	63 89 ± 9 81	67 70 ± 6 43	<0.001	65 72 ± 8 42	65 88 ± 6 15	0.842
SUA (μmol/L)	414.63 ± 107.23	369.01 ± 88.68	<0.001	389.76 ± 89.12	394.24 ± 87.92	0.638
aabSUA	109 (43.6%)	165 (27.5%)	<0.001	59 (33.9%)	69 (39.7%)	0.266
LVDD (mm)	50.73 ± 5.36	47.41 ± 4.06	<0.001	49.12 ± 4.65	48.08 ± 3.97	0.923
CHA_2_DS_2_-VASc score	0.93 ± 0.70	0.84 ± 0.73	0.084	0.89 ± 0.74	0.90 ± 0.70	0.882
LAT/dense SEC	45 (18.0%)	21 (3.5%)	<0.001	27 (15.5%)	9 (5.2%)	0.002

abSUA, abnormal serum uric acid; BMI, body mass index; CHF, congestive heart failure; CAD, coronary artery disease; eGFR, estimated glomerularfiltrationrate; LVEF, left ventricular ejection fraction; LAD, left atrial anteroposterior diameter; LVDD, left ventricular end diastolic diameter; LAT, left atrial thrombosis; SEC, spontaneous echo contrast; PSM, propensity score matching. Age, gender, BMI, current smoking, current alcohol drinking, hypertension, diabetes, CHF, CAD, vascular disease, LVEF, SUA, LVDD and eGFR were used to 1:1 PSM.

### New predictive models

**Figure 1:**
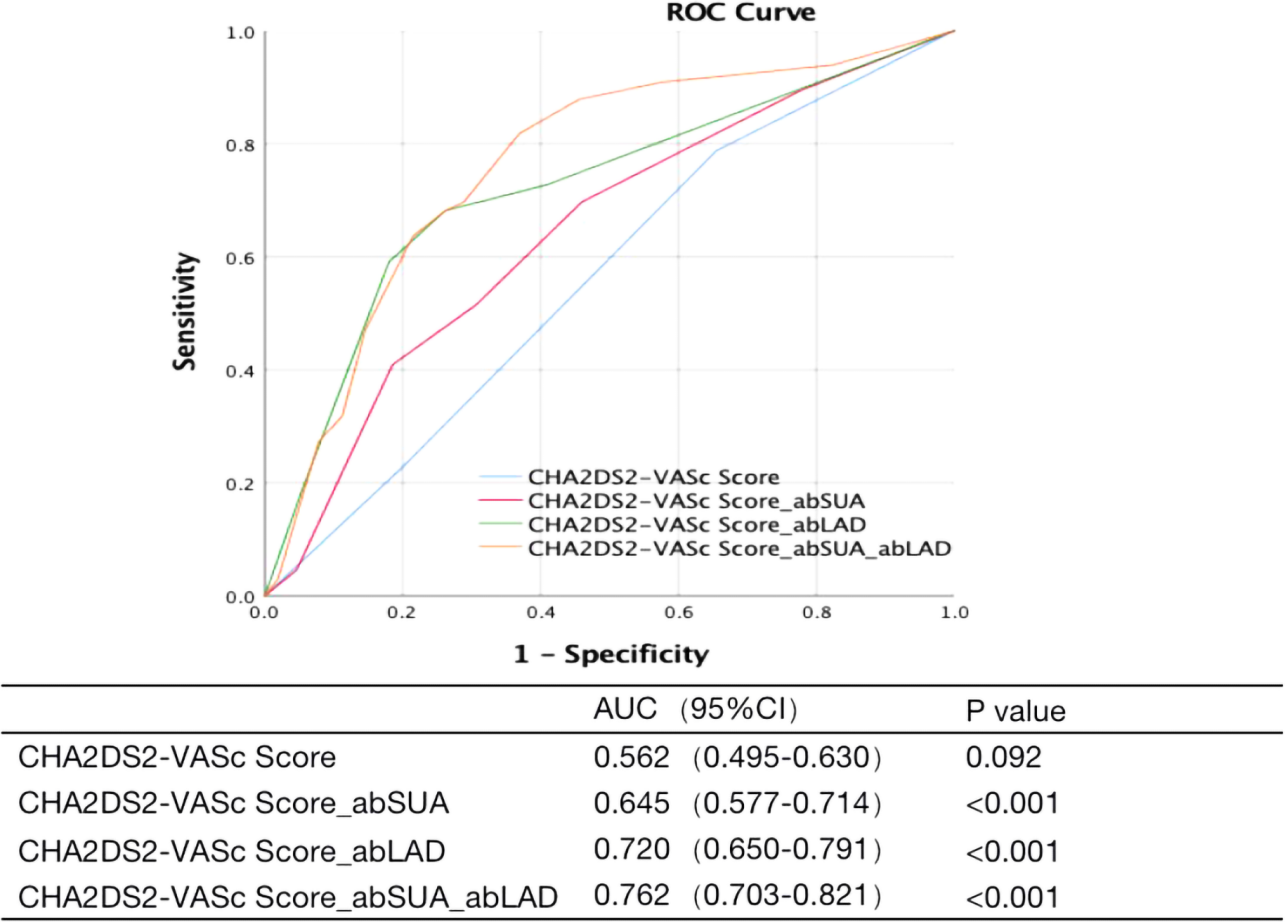
Receiver operating characteristic analysis for LAT/dense SEC prediction model.

Based on the above results, we considered abSUA and abLAD as independent risk factors for LAT/dense SEC. After adding these new risk factors to the patients' existing CHA_2_DS_2_-VASc scores, the area under the ROC curve was used to clarify the predictive effect of the model for LAT/dense SEC in patients with AF. The results showed that the CHA_2_DS_2_-VASc score had no predictive value for the occurrence of LAT/dense SEC in AF patients at low to moderate risk; however, the new prediction model had low to moderate predictive power after the respective addition of abSUA and abLAD. The best predictive value was obtained by adding both abSUA and abLAD (Figure 1).

## Discussion

By retrospectively analyzing the clinical characteristics, both abSUA and abLAD were identified as risk factors beyond the indicators within CHA_2_DS_2_-VASc score for LAT/dense SEC in low to moderate—risk patients with AF. Current guidelines do not explicitly recommend anticoagulants for patients who are deemed low to moderate risk because of their CHA_2_DS_2_-VASc score, but a subset of these patients still develop LAT/dense SEC. It is crucial to assess and predict the thromboembolic risk in this group of patients more comprehensively, as well as to provide better dosing guidance and management.

Many previous studies have confirmed the role of uric acid in the development of cardiovascular events. Lazzeroni et al. found that serum uric acid levels could be used to predict mortality and adverse cardiovascular outcomes in patients undergoing myocardial revascularization and/or heart valve surgery, even after correction for age, sex, hypertension, diabetes mellitus, glomerular filtration rate, and medication (19). Several studies have also confirmed uric acid as a risk factor for AF as well as LAT (20). Other research has likewise suggested that uric acid may contribute to the production of LAT/SEC in patients with AF through the following mechanisms. First, uric acid is the end product of purine metabolism by xanthine oxidase, and the oxygen radicals formed during metabolism increase the level of oxidative stress in atrial tissue. At the same time, uric acid inhibits nitric oxide production by endothelial cells and promotes tissue inflammation, leading to endothelial cell damage (15, 21). Secondly, high uric acid levels activate platelets in the body and promote platelet adhesion and aggregation, which leads to thrombosis (14).

To clarify the relationship between abSUA and LAT/dense SEC in patients with AF, we screened patients with AF who had low to moderate—risk CHA_2_DS_2_-VASc scores for further analysis and found abSUA to be one of their risk factors. The prevalence of LAT/dense SEC was significantly higher in patients with abSUA than in the control group, and this difference persisted after balancing the baseline data between the two groups using PSM.

The association of LA enlargement with increased risk of stroke and thromboembolism in patients with AF was first proposed in the 1980s (22, 23). Over time, more and more studies have demonstrated that LA enlargement is independently associated with an increased risk of stroke in patients with AF, although the endpoint of some studies has been a surrogate marker of stroke on TEE (10, 24–26). Several mechanisms have been proposed to explain the association of LA enlargement with the risk of thromboembolism in patients with AF. AF can lead to structural and functional changes in the atria that are causally related to LA enlargement. The increased internal diameter of the left atrium reduces its blood flow velocity, while the regular contraction of the atrial muscle is replaced by irregular peristalsis in AF. This makes the blood flow prone to vortex formation and even stasis, leading to the retention of organic fractions in the atria. Severe eddies create a shear-like mechanical force on the atrial wall, which in turn damages the endothelia. The slow blood flow causes the atrial endothelium to become hypoxic and necrotic, depriving it of its ability to synthesize and secrete anticoagulant substances, and produces pro-thrombotic substances such as tissue factor, ultimately leading to thrombosis.

In this study, the prevalence of LAT/dense SEC was significantly higher in patients with abLAD than in the control group, and this difference persisted after balancing the baseline information between the two groups using PSM.

The CHA_2_DS_2_-VASc score had no predictive value for the development of LAT/dense SEC in patients with AF who were at low to moderate risk. After the addition of new risk markers using ROC analysis, the results showed that the new model with the respective addition of abSUA and abLAD, especially the combined addition of both abSUA and abLAD, had predictive power for LAT/dense SEC. This finding further suggests that abSUA and abLAD have predictive value for thromboembolic risk in AF patients previously identified as low to moderate risk.

The molecular mechanism of hyperuricemia is a current hot topic of research. Both lifestyle changes and pharmacological interventions can influence uric acid levels. A limitation of this study is that only single measurements of uric acid were included, which provides a somewhat incomplete picture of the long-term uric acid metabolism of patients. In addition, it is unclear whether patients with previous gout or hyperuricemia who were taking uric acid-lowering medication to achieve normal uric acid levels have the same physical impact as patients with naturally-occurring normal uric acid levels. Given the small sample size of this group of patients, a separate analysis could not be performed. The clinical data of this study were obtained from inpatient examination findings of patients. The LAD measured in the hospital was the anterior-posterior LAD, and because of differences in body size and anatomy, the size of the anterior-posterior LAD was often not an accurate assessment of the volumetric size of the left atrium. Some studies have found that the LA volume index better reflects the size of the patient's left atrium (27–29). An association between LAT formation and left auricular morphology and function in AF patients has also previously been found, but given that this study was retrospective, relevant data could not be collected for further analysis (30).

TEE has 100% sensitivity and 99% specificity in detecting LAT as measured by surgical exploration (31), and as such TEE is now considered the gold standard for detecting LAT. In both the present study and previous studies, LAT/dense SEC was present in a certain proportion of patients even at low to moderate risk, and in many centers, TEE is an essential test before invasive treatment in all patients with AF. However, some centers believe that pre-ablation TEE can be reasonably avoided in AF patients without high-risk features. The rate of pre-ablation TEE at Johns Hopkins Hospital was reported to have decreased from 86% in 2010 to 42% in 2015 (32). With the decline in TEE use, risk assessment of LAT in patients with AF becomes critical, especially for patients with AF and a low to moderate—risk score.

In the present study, we found that abSUA and abLAD were risk factors for LAT/dense SEC in AF patients who were previously identified as low to moderate risk according to their CHA_2_DS_2_-VASc scores, suggesting the diagnostic and predictive value of these two commonly used clinical parameters. Future studies and evidence are needed to confirm their correlation.

## Data Availability

The raw data supporting the conclusions of this article will be made available by the authors, without undue reservation.

## References

[1] Wolf PA Abbott RD Kannel WB. Atrial fibrillation as an independent risk factor for stroke: the Framingham study. Stroke. (1991) 22(8):983–8. 10.1161/01.STR.22.8.983 1866765

[2] Kotalczyk A Lip GY Calkins H. The 2020 ESC guidelines on the diagnosis and management of atrial fibrillation. Arrhythm Electrophysiol Rev. (2021) 10(2):65–7. 10.15420/aer.2021.07 34401177 PMC8335854

[3] Gladstone DJ Bui E Fang J Laupacis A Lindsay MP Tu JV Potentially preventable strokes in high-risk patients with atrial fibrillation who are not adequately anticoagulated. Stroke. (2009) 40(1):235–40. 10.1161/STROKEAHA.108.516344 18757287

[4] Perera KS Vanassche T Bosch J Swaminathan B Mundl H Giruparajah M Global survey of the frequency of atrial fibrillation-associated stroke: embolic stroke of undetermined source global registry. Stroke. (2016) 47(9):2197–202. 10.1161/STROKEAHA.116.013378 27507860

[5] Fatkin D Kelly RP Feneley MP. Relations between left atrial appendage blood flow velocity, spontaneous echocardiographic contrast and thromboembolic risk in vivo. J Am Coll Cardiol. (1994) 23(4):961–9. 10.1016/0735-1097(94)90644-0 8106703

[6] Bernhardt P Schmidt H Hammerstingl C Lüderitz B Omran H Patients with atrial fibrillation and dense spontaneous echo contrast at high risk a prospective and serial follow-up over 12 months with transesophageal echocardiography and cerebral magnetic resonance imaging. J Am Coll Cardiol. (2005) 45(11):1807–12. 10.1016/j.jacc.2004.11.071 15936610

[7] Kim YG Shim J Oh SK Lee KN Choi JI Kim YH Risk factors for ischemic stroke in atrial fibrillation patients undergoing radiofrequency catheter ablation. Sci Rep. (2019) 9 (1):7051. 10.1038/s41598-019-43566-z 31065030 PMC6504925

[8] Di Minno MN Ambrosino P Dello Russo A Casella M Tremoli E Tondo C Prevalence of left atrial thrombus in patients with non-valvular atrial fibrillation. A systematic review and meta-analysis of the literature. Thromb Haemost. (2016) 115(3):663–77. 10.1160/th15-07-0532 26607276

[9] Lowe BS Kusunose K Motoki H Varr B Shrestha K Whitman C Prognostic significance of left atrial appendage “sludge” in patients with atrial fibrillation: a new transesophageal echocardiographic thromboembolic risk factor. J Am Soc Echocardiogr. (2014) 27(11):1176–83. 10.1016/j.echo.2014.08.016 25262162

[10] Hamatani Y Ogawa H Takabayashi K Yamashita Y Takagi D Esato M Left atrial enlargement is an independent predictor of stroke and systemic embolism in patients with non-valvular atrial fibrillation. Sci Rep. (2016) 6:31042. 10.1038/srep31042 27485817 PMC4971566

[11] Di Castelnuovo A Veronesi G Costanzo S Zeller T Schnabel RB de Curtis A NT-proBNP (N-Terminal pro-B-type natriuretic peptide) and the risk of stroke. Stroke. (2019) 50(3):610–7. 10.1161/STROKEAHA.118.023218 30786848

[12] He H Guo J Zhang A. The value of urine albumin in predicting thromboembolic events for patients with non—valvular atrial fibrillation. Int J Cardiol. (2016) 221:827–30. 10.1016/j.ijcard.2016.07.145 27434352

[13] Ning W Li Y Ma C Qiu L Yu B The refinement of risk stratification for atrial thrombus or spontaneous echo contrast in non-valvular atrial fibrillation. Int Heart. (2017) 58:885–93. 10.1536/ihj.16-444 29151480

[14] Mustard JF Murphy EA Ogryzlo MA Smythe HA. Blood coagulation and platelet economy in subjects with primary gout. Can Med Assoc J. (1963) 89:1207–11. 14084698 PMC1922159

[15] Kanabrocki EL Third JL Ryan MD Nemchausky BA Shirazi P Scheving LE Circadian relationship of serum uric acid andnitric oxide. JAMA. (2000) 283:2240–1. 10.1001/jama.283.17.2235 10807381

[16] Yan S Li Q Xia Z Yan S Wei Y Hong K Risk factors of thromboembolism in non-valvular atrial fibrillation patients with low CHA_2_DS_2_-VASc score. Medicine (Baltimore). (2019) 98(8):e14549. 10.1097/MD.0000000000014549 30813164 PMC6408143

[17] Yao Y Shang MS Gao LJ Zhao JH Yang XH Liu T Elevated homocysteine increases the risk of left atrial/left atrial appendage thrombus in non-valvular atrial fibrillation with low CHA_2_DS_2_-VASc score. Europace. (2018) 20(7):1093–8. 10.1093/europace/eux189 28637244

[18] Kimura T Takatsuki S Inagawa K Katsumata Y Nishiyama T Nishiyama N Anatomical characteristics of the left atrial appendage in cardiogenic stroke with low CHADS_2_ scores. Heart Rhythm. (2013) 10(6):921–5. 10.1016/j.hrthm.2013.01.036 23384894

[19] Lazzeroni D Bini M Camaiora U Castiglioni P Moderato L Bosi D Serum uric acid level predicts adverse outcomes after myocardial revascularization or cardiac valve surgery. Eur J Prev Cardiol. (2018) 25(2):119–26. 10.1177/2047487317744045 29164926

[20] Tang RB Dong JZ Yan XL Du X Kang JP Wu JH Serum uric acid and risk of left atrial thrombus in patients with non-valvular atrial fibrillation. Can J Cardiol. (2014) 30(11):1415–21. 10.1016/j.cjca.2014.06.009 25442440

[21] Korantzopoulos P Kolettis TM Galaris D Goudevenos JA The role of oxidative stress in the pathogenesis and perpetuation of atrial fibrillation. Int J Cardiol. (2007) 115(2):135–43. 10.1016/j.ijcard.2006.04.026 16764958

[22] Caplan LR D'Cruz I Hier DB Reddy H Shah S Atrial size, atrial fibrillation, and stroke. Ann Neurol. (1986) 19(2):158–61. 10.1002/ana.410190208 3963758

[23] Aronow WS Gutstein H Hsieh FY. Risk factors for thromboembolic stroke in elderly patients with chronic atrial fibrillation. Am J Cardiol. (1989) 63(5):366–7. 10.1016/0002-9149(89)90349-4 2783633

[24] Ogata T Matsuo R Kiyuna F Hata J Ago T Tsuboi Y Left atrial size and long-term risk of recurrent stroke after acute ischemic stroke in patients with nonvalvular atrial fibrillation. J Am Heart Assoc. (2017) 6(8):e006402. 10.1161/JAHA.117.006402 28862939 PMC5586470

[25] Providência R Botelho A Trigo J Quintal N Nascimento J Mota P Possible refinement of clinical thromboembolism assessment in patients with atrial fibrillation using echocardiographic parameters. Europace. (2012) 14(1):36–45. 10.1093/europace/eur272 21868410

[26] Faustino A Providência R Barra S Paiva L Trigo J Botelho A Which method of left atrium size quantification is the most accurate to recognize thromboembolic risk in patients with non-valvular atrial fibrillation? Cardiovasc Ultrasound. (2014) 12:28. 10.1186/1476-7120-12-28 25052699 PMC4121510

[27] Ayirala S Kumar S O'Sullivan DM Silverman DI Echocardiographic predictors of left atrial appendage thrombus formation. J Am Soc Echocardiogr. (2011) 24(5):499–505. 10.1016/j.echo.2011.02.010 21440414

[28] Doukky R Khandelwal A Garcia-Sayan E Gage H External validation of a novel transthoracic echocardiographic tool in predicting left atrial appendage thrombus formation in patients with non-valvular atrial fibrillation. Eur Heart J Cardiovasc Imaging. (2013) 14(9):876–81. 10.1093/ehjci/jes313 23291395

[29] Osranek M Bursi F Bailey KR Grossardt BR Brown RD Jr Kopecky SL Left atrial volume predicts cardiovascular events in patients originally diagnosed with lone atrial fibrillation: three-decade follow-up. Eur Heart J. (2005) 26(23):2556–61. 10.1093/eurheartj/ehi483 16141257

[30] Qamruddin S Shinbane J Shriki J Naqvi TZ. Left atrial appendage: structure, function, imaging modalities and therapeutic options. Expert Rev Cardiovasc Ther. (2010) 8(1):65–75. 10.1586/erc.09.161 20014936

[31] Manning WJ Weintraub RM Waksmonski CA Haering JM Rooney PS Maslow AD Accuracy of transesophageal echocardiography for identifying left atrial thrombi. A prospective, intraoperative study. Ann Intern Med. (1995) 123(11):817–22. 10.7326/0003-4819-123-11-199512010-00001 7486462

[32] Balouch M Gucuk Ipek E Chrispin J Bajwa RJ Zghaib T Berger RD Trends in transesophageal echocardiography use, findings, and clinical outcomes in the era of minimally interrupted anticoagulation for atrial fibrillation ablation. JACC Clin Electrophysiol. (2017) 3(4):329–36. 10.1016/j.jacep.2016.09.011 29759444

